# The impact of hypoxia on intestinal epithelial cell functions: consequences for invasion by bacterial pathogens

**DOI:** 10.1186/s40348-016-0041-y

**Published:** 2016-03-22

**Authors:** Nathalie E. Zeitouni, Sucheera Chotikatum, Maren von Köckritz-Blickwede, Hassan Y. Naim

**Affiliations:** Department of Physiological Chemistry, University of Veterinary Medicine Hannover, Hannover, Germany; Research Center for Emerging Infections and Zoonoses (RIZ), University of Veterinary Medicine Hannover, Hannover, Germany

**Keywords:** Oxygen, Invasion, β1 integrin, Infection, HIF-1α, Intestine

## Abstract

The maintenance of oxygen homeostasis in human tissues is mediated by several cellular adaptations in response to low-oxygen stress, called hypoxia. A decrease in tissue oxygen levels is initially counteracted by increasing local blood flow to overcome diminished oxygenation and avoid hypoxic stress. However, studies have shown that the physiological oxygen concentrations in several tissues are much lower than atmospheric (normoxic) conditions, and the oxygen supply is finely regulated in individual cell types. The gastrointestinal tract has been described to subsist in a state of physiologically low oxygen level and is thus depicted as a tissue in the state of constant low-grade inflammation. The intestinal epithelial cell layer plays a vital role in the immune response to inflammation and infections that occur within the intestinal tissue and is involved in many of the adaptation responses to hypoxic stress. This is especially relevant in the context of inflammatory disorders, such as inflammatory bowel disease (IBD). Therefore, this review aims to describe the intestinal epithelial cellular response to hypoxia and the consequences for host interactions with invading gastrointestinal bacterial pathogens.

## Physiological oxygen concentrations

**Fig. 1:**
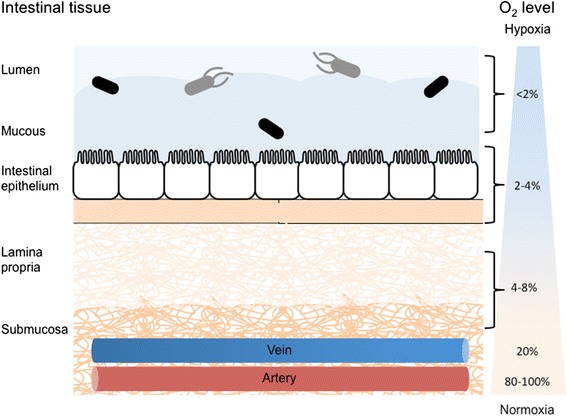
Schematic of gut oxygen gradient. The intestine faces daily fluctuations in blood flow and a steep oxygen gradient is present, extending from the highly vascularized and oxygenated subepithelial mucosa (4–8 %), across the epithelial and mucous layer (2–4 %), and into the severely oxygen-deficient lumen (<2 %). Arterial blood oxygen content is approximated as 80–100 % while venal blood oxygen content is approximated as 20 % [1, 4, 5]

Researchers have aptly described three terms that characterize the different oxygen levels to which cells are exposed: “normoxia” that denotes atmospheric oxygen content, “physioxia” that signifies biologically sufficient oxygen concentration in tissues, and “hypoxia” that represents oxygen concentrations less than normal, indicating an oxygen deficit [1]. Hypoxia has a substantial effect on several cellular processes and has been shown to influence the pathogenesis of several diseases including gastrointestinal disorders, tumors, and cardiovascular diseases [2]. Within the human body, the different tissues are supplied with varying concentrations of oxygen, depending on their specific metabolic demands. Therefore, a state of oxygen concentration that is physiologically lower than atmospheric does not necessarily indicate a deficit in oxygen supply or the existence of hypoxic stress [3]. The intestinal tissue, for instance, when measured with a multiwire platinum surface electrode, showed an average oxygen concentration of around 7 %, at the serosal side of the small bowel [1]. Moreover, the intestine faces daily fluctuations in perfusion, with higher blood and oxygen flow after food intake as compared to fasting levels [4]. Furthermore, due to its distinctive structure within the body, with the highly vascularized and oxygenated subepithelial mucosa on one side and the severely oxygen deficient lumen on the other side, the gastrointestinal (GI) tract is characterized by a steep oxygen gradient across the epithelial layer [4]. Non-invasive measurement of tissue oxygen partial pressure (pO_2_) by electron paramagnetic resonance (EPR) oximetry showed oxygen levels ranging from 8 % in the small intestinal wall to around 3 % at the villus tip and less than 2 % in the intestinal lumen [5]. A basic schematic of this gradient is displayed in Fig. 1. Considering these diverse oxygen levels and the daily fluctuations experienced in tissues, these conditions of physiological oxygenation or physiological hypoxia were termed physioxia [3]. In addition to studying the gastrointestinal environment and the host-pathogen interactions that occur in this tissue, it is important to find an adequate cell culture model that on the one hand closely mimics physiological relevant conditions and on the other hand facilitates experimental procedures. A recent in vitro study on Caco-2 cultures, using non-invasive optical sensing techniques, showed pO_2_ values of around 3 % in the differentiated cells grown in a traditional tissue culture incubator, somewhat mimicking the in vivo physiological oxygen conditions [6]. These data further underline the significance of examining the effects of hypoxic adaptation on host-pathogen interactions in both in vivo and in vitro settings.

## Hypoxia during infections and inflammation

Hypoxic stress, that occurs when cellular oxygen demand is higher than its supply, is a commonplace in tissues faced with infection and inflammation [7, 8]. There are many factors that result in this oxygen deficit, including the demands of innate immune cells, such as neutrophils and macrophages that are recruited to the site of infection as well as those of invading pathogens that also consume oxygen [9, 10]. These increased oxygen demands, in addition to the requirements of the resident cells of the infected tissue, can cause a severe drop in available oxygen levels, resulting in a state of hypoxia. Infiltrating neutrophils play an important role in the host response to inflammation and result in depletion of oxygen, transcriptional changes, and aberrant vascularization [10]. Recruited polymorphonuclear neutrophils (PMNs) rapidly generate reactive oxygen species, mediated by a powerful oxidative burst, thus immensely increasing oxygen consumption. Activated PMNs elicit an almost 50-fold increase in oxygen demands and thus contribute to the hypoxic conditions in the inflamed tissue [7, 8].

**Fig. 2:**
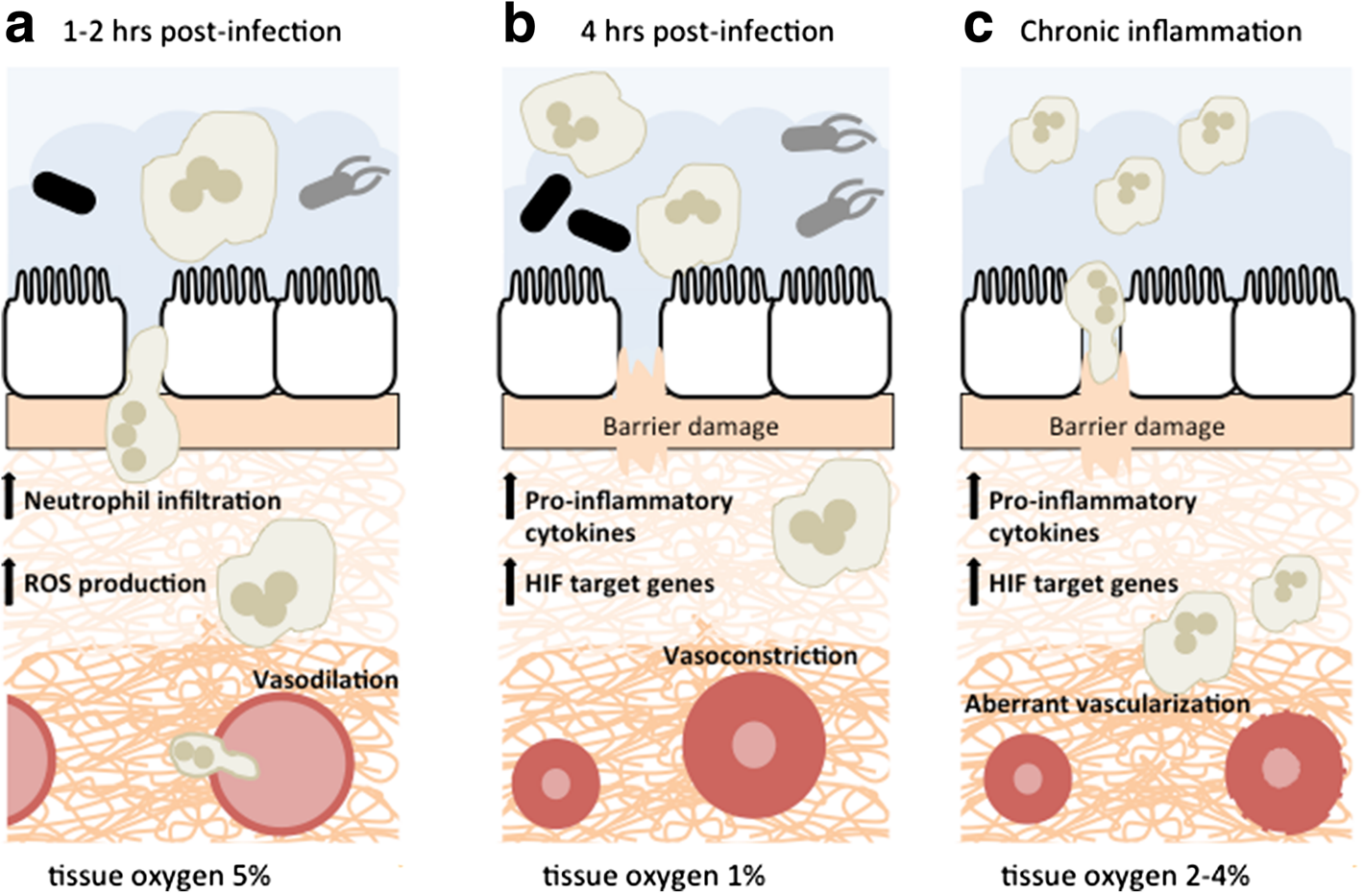
Intestinal tissue oxygen levels after infection or chronic inflammation. **a** One hour after infection, infiltration by neutrophils causes an increase in ROS production and subsequent decrease in oxygen levels, from 7 % to almost 5 %. Vasodilators are released to promote microvessel perfusion. Epithelial barrier is intact, and bacterial spread is contained. **b** As the infection progresses, pro-inflammatory cytokines are released and more PMNs are recruited to the tissue further decreasing local oxygen levels to less than 1 %. The epithelial layer is disrupted, and blood vessels become constricted because of clotting. Several hypoxia-dependent genes are upregulated. **c** In tissues with chronic inflammation, infiltrating neutrophils also lead to depletion of oxygen. Transcriptional changes in hypoxia-dependent genes along with aberrant vascularization create a severe hypoxic environment (2–4 %) [7, 8, 11]

A state of hypoxia resulting from infections has been shown in vivo in several studies. Infection of renal tubules with uropathogenic *Escherichia coli* (UPEC) in rats resulted in a severe drop in pO_2_ from 7 % in uninfected tissues to 5 % O_2_ 1 h post-infection and finally to almost 0 % O_2_ within 4 h of infection, as measured by Clark-type microelectrodes [11]. This dramatic decrease in pO_2_ may be partially due to the recruitment of activated leukocytes that congest the capillaries and release reactive oxygen species as they translocate towards the site of infection [11]. Furthermore, tissues that subsist under conditions of chronic inflammation are shown to have a reduction in blood supply and a consequent loss of adequate oxygenation [9]. During inflammation and hypoxia, angiogenesis is induced to compensate for poor oxygenation; however, this may result in aberrant vasculature and contribute to the pathogenesis of chronic inflammation [7]. In IBD, vascular endothelial growth factor (VEGF)-dependent angiogenesis and increased production of vasoconstrictors were shown to result in abnormal microvasculature [8]. Figure 2 summarizes the events that lead to decreased oxygen levels in the intestinal tissue during an infection and chronic inflammation.

The human gut is host to a large number of commensal bacteria that inhabit the lumen and epithelial mucosa of the lower intestine and has developed productive relationships with its microbiota; however, it remains highly vigilant against invading pathogens [12]. In cases when the balance of the normal flora is upset, or if the intestinal barrier is breached, infection can occur from invading pathogens or from overgrowth of endogenous pathogens [12]. Indeed, invasive enteropathogenic bacteria, including *Salmonella*, *Shigella*, and *Yersinia*, cause considerable damage to the mucosal layer and the intestinal epithelial cells as well as the lamina propria [12]. Enterocolitis, as the most common presentation of *Yersinia enterocolitica* infection, occurs primarily in young children, with a mean age of 24 months. The incubation period is 4–6 days, typically with a range of 1–14 days. Most cases are self-limited. However, concomitant bacteremia may occur in 20–30 % of infants younger than 3 months [13]. Besides the physical and structural damage that occur during those intestinal infections, many of the intestinal pathogens induce the expression of inflammatory and chemoattractive cytokines that collectively raise an immune response [14]. Therefore, it is a point of interest to investigate the fate of the epithelial layer of the intestinal tissue during a bacterial infection under hypoxia and whether the cellular adaptation mechanisms offer the host any protective or defensive advantages. Understanding the protective host response under hypoxia might finally help to develop new prophylactic or therapeutic strategies that might be supportive for the host during cellular stress response.

## Transcriptional response to hypoxia

Living organisms have developed rather efficient mechanisms to maintain cellular homeostasis and to circumvent stressful conditions. At the cellular level, many genes are involved in the adaptation processes either in a regulatory capacity or in a functional manner. One very well-characterized regulator of the cellular response to low oxygen levels is the transcription factor hypoxia inducible factor 1 (HIF-1). HIF-1 has been found to bind to and induce the expression of several genes whose products promote erythropoiesis and angiogenesis and are involved in glucose transport and metabolism, thus initiating the cellular adaptation response to hypoxic stress [15, 16]. HIF-1 is a transcription factor consisting of two subunits: the oxygen regulated alpha (α) and a constitutively expressed beta (β) subunit, also known as the aryl hydrocarbon receptor nuclear translocator (ARNT) [17]. HIF-1α protein is a global regulator of the energy homeostasis and cellular adaptation to hypoxia, and its stability is tightly regulated by the cellular oxygen concentration [18]. During conditions of adequate oxygenation, or normoxia, HIF-1α is rapidly degraded by binding of the von Hippel-Lindau tumor suppressor protein (pVHL) that subsequently targets it for ubiquitination and proteosomal degradation [19]. This process is mediated by oxygen- and iron-dependent prolyl hydroxylases (PHDs) that transfer a hydroxyl group onto two proline residues (P402 and P564) allowing for binding to pVHL [19]. Under hypoxic conditions, HIF-1α rapidly accumulates due to the interruption of its degradation pathway by inhibition of the oxygen-dependent hydroxylation [20].

Several HIF-1 target genes have been shown to mediate a protective effect on the mucosal layer through the upregulation of CD55, ecto-50 nucleotidase, MUC-3, intestinal trefoil factor, and P-glycoprotein [21]. This enhanced expression of barrier-protective genes may present an obstacle to invasive gastrointestinal bacteria that seek to invade by paracellular translocation across the epithelial layer, like *Vibrio cholera* and *Clostridium difficile* [22]*.* Furthermore, nuclear factor-kappa B (NF-κB), a central regulator of innate immunity and inflammatory processes, is activated in hypoxia, both in vitro and in vivo [23]. A complex connection exists between HIF-1 and NF-κB; both transcription factors have several target genes in common, and NF-κB activation can stabilize HIF-1α, yet HIF-1 can repress NF-κB activity during inflammation [21]. However, studies have also implicated HIF-independent NF-kB-mediated pathways in the host response at sites of inflammation, where NF-kB is required for the maintenance of epithelial barrier integrity [8, 21]. Conditional deletion of NF-kB in intestinal epithelial cells in mice led to an increased susceptibility to colitis in a murine model [24]. It has therefore been suggested that hypoxia regulates inflammatory responses through the activation of NFκB signaling pathway in a multi-factorial process [25]. This hypoxia-mediated induction of inflammatory responses may provide a better defense against invading pathogens.

## Cellular adaptation to hypoxia

### Bacterial invasion mechanisms: trigger or zipper

The intestinal epithelial layer is a crucial barrier against invading pathogens and resistance to infection; however, it is also the point of entry for many bacteria [22]. Since epithelial cells are non-phagocytic in nature, pathogens need to actively induce their own entry into host cells [26]. Thus, two major pathogen-mediated entry mechanisms have been identified: the binding of bacterial adhesins to host cell receptors (zipper mechanism) and the injection of bacterial effector proteins into the host cell cytosol (trigger mechanism), both of which are followed by cell signaling pathways that result in cytoskeletal remodeling and subsequent engulfment [26]. Among the common gastrointestinal pathogens, *Y. enterocolitica* utilizes the zipper mechanism while *Shigella flexneri* has been known to employ the trigger mechanism to invade host cells [27]. *Pseudomonas aeruginosa*, a generally opportunistic bacterium associated with nosocomial infections, has been increasingly found in the gastrointestinal tract of hospitalized patients [28]. *P. aeruginosa* internalizes into host epithelial cells by the trigger mechanism, using one of its injected effector proteins, ExoS that binds to the mammalian factor FXYD3 [29]. Interestingly, these abovementioned three intestinal pathogenic bacteria exhibit decreased levels of internalization into intestinal epithelial cells (for *Y. enterocolitica* and *S. flexneri*) or pulmonary epithelial cells (for *P. aeruginosa)* pre-incubated under 1 % O_2_ for 24 h [30, 31]. In light of these results, understanding the cellular effects of hypoxia on epithelial cells, e.g., membrane alterations, cytoskeletal rearrangements, or endoplasmatic reticulum (ER) stress during infections, offers a new potential to find targets for pharmacological interference. Thus, we sought to correlate this phenomenon of decreased internalization with the various hypoxia-induced cellular adaptations that have been published so far. However, we have to highlight the fact that two other well-known pathogens, *Listeria monocytogenes* and *Salmonella typhimurium* that internalize by the zipper and trigger mechanisms, respectively, exhibited increased entry into HT-29 enterocyte cells when cells are pre-incubated under 10 and 5 % hypoxia for 21 days [32]. The specific mode of entry of gastrointestinal pathogens, their effector proteins, and host receptors or targets as well as the proposed mechanism for hypoxia-mediated changes in internalization is presented in Table 1.

**Table 1:** Main gastrointestinal pathogens and their specific mode of entry

Bacterial invasion	Pathogen	Host receptor/target	Mechanism	Internalization under hypoxia	Potential mechanism of hypoxia-induced changes
*Zipper mechanism*	*Yersinia* (invasin)	β1-integrin	Signaling from Rac1 to Arp2/3 [37]	↓ [39]	Decreased receptor protein expression, reduced glycosylation and mislocalization in lipid rafts [39]
	*Listeria* (InlA)	E-cadherin	Ligase Hakai recruitment, clathrin endocytosis, and activation of Arp2/3 actin complex [53]	↑ [32]	Elevated expression of barrier protection genes, more increased levels of E-cadherin [54]
	*Listeria* (InlB)	Met (hepatocyte growth factor receptor)	Activation of Met and PI-3-kinase-mediated signaling [53]	↑ [32]	Increased expression of growth factor receptors [55]
*Trigger mechanism*	*Pseudomonas* (ExoS)	Mammalian factor FXYD3	Impairment of function of tight junctions [29]	↓ [31]	Increased barrier protection, more stable adherens, and tight junctions [54]
	*Shigella* (IpaC)	Cdc42, Rac1, Rho	Activation of target, membrane ruffling [73]	↓[30]	Cytoskeleton rearrangements hinder membrane ruffling [40]
	*Salmonella* (SipA, SipC)	Phosphatidylinositol 4,5-bisphosphate PtdIns (4,5) P2	Phosphoinositide signaling; membrane ruffling and formation of macropinosomes [41]	↑ [32]	Changes in membrane lipid composition [40]

### Membrane alterations

One region of the plasma membrane that poses great interest to host pathogen interactions is lipid rafts. Lipid rafts are ordered liquid domains rich in sphingolipids and cholesterol and segregated from less-ordered liquid domains composed of mainly unsaturated phospholipids [33]. Cell signaling, intracellular membrane transport, cell adhesion, and host-pathogen interactions are among the cell processes regulated by lipid rafts [34]. Therefore, any chemical and physical perturbations of plasma membrane structure or composition may have a dramatic effect on cellular processes that are associated with lipid rafts. In fact, hypoxic exposure leads to the selective remodeling of membrane lipids and proteins, more specifically to an increase in saturated fatty acid content due to the inability to perform β-oxidation in oxygen-limited conditions while amounts of phospholipids and free cholesterol remain unchanged [35]. In alveolar cells, mild hypoxia results in a significant increase in the cholesterol to phospholipids ratio causing a decrease in membrane fluidity, with no significant increase in lipid peroxidation [36]. This selective lipid enrichment and decrease in membrane fluidity under hypoxia is suggested as an adaptation response to regulate the function of membrane-bound proteins and their localization by decreasing endocytosis [35]. Furthermore, when membrane composition is altered, membrane-associated proteins are also most likely affected. The protein marker of caveolae, caveolin 1 (Cav-1), reveals reduced levels in the lipid microdomains while the total content of this protein the membranes remains unchanged, thus indicating a redistribution within the membrane [36]. These studies were performed in various types of cells, however, and not much is known about membrane alterations in intestinal epithelial cells. Considering that the main point of contact between pathogens and intestinal host cells is at the plasma membrane, it is possible that alterations in membrane-associated receptors may protect against bacterial invasion. Host β1 integrins, that are the main receptors for *Y. enterocolitica*, are lipid raft-associated proteins that require these platforms for clustering as well as recycling [37, 38]. In the absence of anchored and clustered receptors at the cell surface, due to hypoxia-mediated alterations, bacterial attachment and internalization may be greatly hindered, thus providing the host cells with protection against invading pathogens. In fact, we have shown a significant reduction in brush border membrane enrichment of β1 integrins under hypoxia, thus severely reducing *Y. enterocolitica* internalization into Caco-2 cells [39]. Furthermore, studies have shown that after 5-h incubation under 1 % O_2_, phosphatidylinositol activity is increased in hepatoma cells [40]. Since phosphatidylinositol is used by *S. typhimurium* to internalize into host cells, its accumulation under hypoxia may explain the increased pathogen entry [32]. On the other hand, *S. flexneri* effectors deplete phosphatidylinositol from the plasma membrane in order to limit membrane cytoskeletal interactions and facilitate entry into host cells [41]. An increase in membrane phosphatidylinositol levels may be the reason why Shigella entry into host cells is hindered under hypoxia [30].

### Cytoskeletal rearrangements

Another key aspect of the cellular response to hypoxia is cytoskeletal adaptation. Studies have reported hypoxia-induced disorganization of the cytoskeletal network by disrupting F-actin filaments and by excessive cleavage of α-spectrin, an apical protein, that binds to the actin cytoskeleton and sodium transport proteins [42]. Furthermore, hypoxia has been shown to have a distinct effect on epithelial cells by disrupting the actin cytoskeleton and tight junctions, by mislocalization of occludin and reduction of the zonula occludens 1 [42, 43]. Furthermore, hypoxia regulates the Rho guanosine triphosphatases (GTPases) that modulate the activity of actin-binding proteins, by inhibiting their isoprenylation and thus resulting in decreased actin polymerization and eventually in impaired endocytosis [35]. Since many invasive pathogens, such as *Shigella* and *Yersinia* species, hijack the host cytoskeletal system in order to internalize into their target cells, any alterations in the cytoskeletal activity and structure can hinder this internalization process [27]. Oxygen-dependent modifications of the host cytoskeleton also significantly affect the paracellular permeability, intracellular transport, and the general endocytic uptake of particles in alveolar epithelial cells [42]. Since some of these bacteria are internalized into the intestinal epithelial cells via some form of endocytosis or membrane invagination, fragments of host plasma membrane will form the pathogen-containing compartments. Therefore, alterations in the host endocytic process caused by oxygen deficiency would also affect these compartments and ultimately influence intracellular bacterial survival [44].

### Endoplasmic reticulum stress

The endoplasmic reticulum (ER) is responsible for protein translocation, folding, post-translational modifications, and finally, protein delivery to their proper target sites [45]. Under stressful conditions such as inflammation or infection, ER homeostasis is disturbed, causing the accumulation of misfolded or unfolded proteins in the ER lumen and ultimately leading to the induction of the unfolded protein response (UPR) [45]. The UPR consists of several steps, including (1) lowered protein biosynthesis and translocation into the ER, (2) increased expression of chaperones to aid in protein folding, (3) degradation of unfolded proteins, and finally, (4) apoptosis that eventually leads to pathogenic phenotypes [45]. Hypoxia has been shown to induce ER stress and can lead to the initiation of UPR, which in turn increases the transcription of pro-angiogenic genes such as VEGF by enhancing HIF-1α activity [46].

**Fig. 3:**
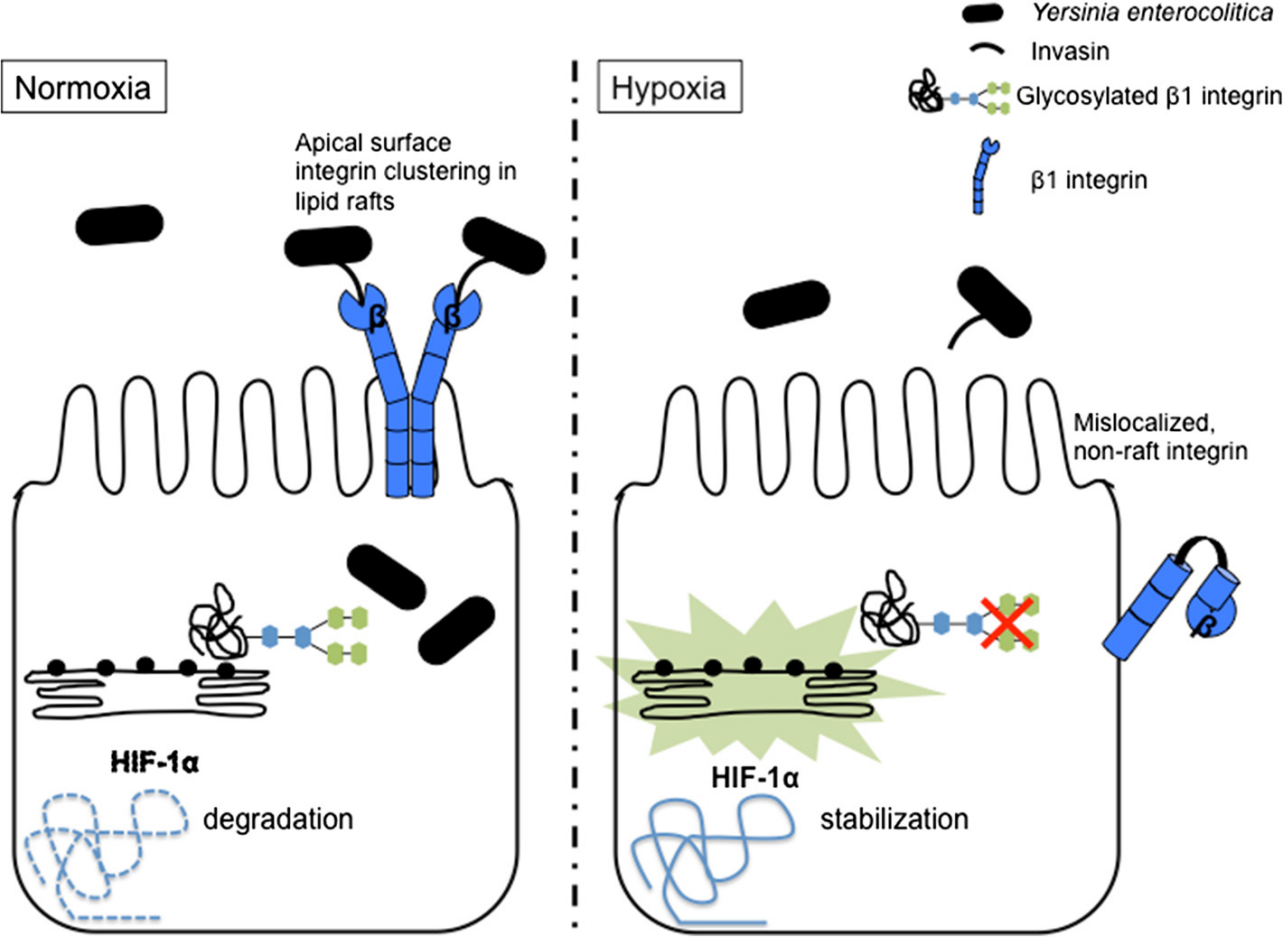
Hypothesized cellular adaptation models. Under normoxia, β1 integrins are properly glycosylated in the ER, are subsequently trafficked to the cell surface, and associate with lipid rafts. This allows binding to *Yersinia enterocolitica* invasin and bacterial internalization into host intestinal epithelial cell. Under hypoxia, HIF-1/hypoxia-induced ER stress and membrane alterations result in improper glycosylation and mislocalization of β1 integrins, thus reducing binding to and internalization of *Y. enterocolitica*

A large number of proteins are glycosylated in the ER not only to ensure proper trafficking to the cell surface in general [47] but also to differentially sort proteins to either the apical or basolateral membrane in polarized epithelial cells [48]. When ER stress is induced under hypoxia, and protein processing is altered, this may result in faulty trafficking and mislocalization of proteins that would normally act as cell surface receptors. The β1 integrin host receptors are indeed heavily glycosylated [49], and any alterations in the processing of these proteins may limit their availability at the cells surface, ultimately preventing bacterial internalization into host epithelial cells. Interestingly, many post-translational modifications to β integrins have been described after exposure to hypoxia, including uncontrolled cleavage by the protease calpain resulting in severely reduced protein levels at the surface of renal epithelial cells [50]. Furthermore, hypoxia significantly alters β1 integrin activation by affecting its maturation, glycosylation, and localization, thus interfering with various functions of this receptor and disrupting downstream signaling cascades [51, 52]. It is therefore hypothesized that hypoxia-mediated modifications to β1 integrins may obstruct *Y. enterocolitica* internalization into intestinal epithelial cells, as displayed in the model in Fig. 3. Transcriptional changes as well as post-translational modifications may also affect the internalization of *L. monocytogenes* and *P. aeruginosa. L. monocytogenes* internalizes into host cells via InlA and InlB that target E-cadherin and Met, the hepatocyte growth factor receptor, respectively [53]. Both of these host receptors displayed increased expression under hypoxia, possibly resulting in increased *L. monocytogenes* entry into enterocytes [54, 55]. *P. aeruginosa* entry utilizes the host receptor FXYD3 that both regulates and co-localizes with sodium-potassium ATPases [29]. Under hypoxia, Na-K-ATPase expression is decreased [56] and FXYD3 was found to be downregulated in breast and lung epithelial tumors [57, 58], all of which may play a role in the decreased internalization of *P. aeruginosa* into epithelial cells [31].

## Mucosal immunity

The first barrier that microorganisms encounter in the intestine is a thick mucosal layer overlying the epithelial cells that provides protection against invading pathogens and other chemical or mechanical threats, composed mainly of glycoproteins called mucins [59]. During infections, mucins are commonly associated with pathogen adherence, and both *S. typhimurium* and *Y. enterocolitica* virulent strains were shown to specifically bind to mucins in the intestinal tract [60, 61]. Mucin expression can be increased by various cues, such as exposure to microbes, pro-inflammatory cytokines, and resident macrophages, thus providing a link between innate mucosal immunity, and inflammatory responses [59]. Increased expression of Muc 2, a major intestinal mucin, was shown to protect against infectious colitis by limiting *Citrobacter rodentium* translocation across the intestinal epithelial layer in mice [62]. Interestingly, hypoxia increases the expression of Muc 1 and Muc 2 in a HIF-1α-dependent manner in renal and peritoneal tumors, respectively [63, 64]. Whether this hypoxia-mediated increase in mucins aids in the entrapment of invading pathogs or serves to strengthen the epithelial barrier remains unclear. Besides transcriptional regulation of mucins, alterations in mucin glycosylation have been shown to occur, thus affecting microbial adhesion and circumventing mucus degradation by microbes [59]. As we have discussed in this review, hypoxia-induced ER stress can lead to alterations in glycosylation patterns, suggesting a potential role for hypoxia in mucin response to infections and their role in pathogen clearance.

## Relevance in the context of inflammatory bowel diseases

The GI tract has been described to be in a state of constant, low-grade inflammation associated with hypoxia, with intestinal epithelial cells playing a pivotal role in mucosal immunity in response to this inflammation and in maintaining homeostasis [8]. Chronic gastrointestinal inflammatory conditions, such as Crohn’s disease and ulcerative colitis, are characterized by exaggerated inflammatory responses to the luminal microbiome and are aggravated by the resulting epithelial barrier dysfunction [65].

Interestingly, HIF-1α was found to be highly expressed in epithelial cells from both Crohn’s disease and ulcerative colitis patients [66]. The role of HIF-1α and its activated pathways was revealed to be a protective one, with suggested mechanisms that include improved barrier protection and prevention of epithelial cell apoptosis [21, 67]. Furthermore, conditional knockout of HIF-1α expression in intestinal epithelial cells exacerbated barrier injury and resulted in more severe symptoms in a murine colitis model [54]. A similar aggravation in inflammatory damage was seen in *C. difficile* toxin-induced colitis in mice lacking intestinal epithelial HIF-1α [68]. In contrast, increasing HIF-1α by pharmacological inhibition of its degradation substantially reduced the extent of injury caused by inflammatory damage in these colitis models [67, 68].

In light of increasing antibiotic resistance, novel approaches to treatment of infections are needed, and methods for boosting the host defense are currently being explored [69]. Because of its implication in the hypoxia-induced modulation of the immune response, HIF-1α has been considered as a possible novel therapeutic target [70]. Pharmacological manipulation of HIF-1α significantly improved the ability of keratinocytes to fight against skin infections [71]. Treatment with a HIF-1α agonist boosts the innate immune response of the intestinal epithelium in a murine colitis model [72].

These data strongly suggest that HIF-1α is a promising candidate for use as a therapeutic agent for the treatment of several pathological conditions. The ability of HIF-1α to boost the innate immune cells is a very attractive feature that can be effective in the treatment of multi-drug resistant bacterial infections or in immunocompromised patients [69]. Since the pharmacological stabilization of HIF-1α results in the increase of a number of antimicrobial host agents, the chances for the development of bacterial resistance are greatly diminished [70].

Ultimately, it is important to emphasize the relevance of studying hypoxia and the pathways involved in the adaptation to oxygen stress in the context of gastrointestinal perturbations and the host cell endeavors to maintain homeostasis.
